# Why are extreme precipitation events becoming more frequent in a warming world?

**DOI:** 10.1016/j.fmre.2024.11.009

**Published:** 2024-11-20

**Authors:** Wei Huang, Tingting Xie, Lingxin Huang, Shuo Hao, Yanwu Duan

**Affiliations:** a Faculty of Arts and Sciences, Beijing Normal University, Zhuhai 519085, China; b College of Water Resources and Architectural Engineering, Tarim University, Xinjiang 843300, China; c Key Laboratory of Western China's Environmental Systems (Ministry of Education) College of Earth and Environmental Sciences, Lanzhou University, Lanzhou 730000, China; d Alpine Paleoecology and Human Adaptation Group, State Key Laboratory of Tibetan Plateau Earth System, Environment and Resources, Institute of Tibetan Plateau Research, Chinese Academy of Sciences, Beijing 100101, China

Under the combined effects of climate change and human activities, there is a significant increase in extreme events, which can substantially impact human productivity, society, and ecosystems in a short period. Extreme precipitation events have garnered widespread attention due to their greater socio-economic impact. While climate models predict that global precipitation will become more variable with global warming, there is still a lack of observational evidence. Additionally, systematic conclusions on precipitation variability's cross-temporal and spatial scales are insufficient. There is also insufficient evidence regarding its mechanisms of influence and the significant role of human activities.

Wenxia Zhang and colleagues at Institute of Atmospheric Physics of the Chinese Academy of Sciences studied all publicly available international daily precipitation observational data since 1900, which was recently published in Science [1]. They used precipitation variability (temporal standard deviation) to represent global changes and their impact mechanisms, making it a useful indicator for extreme precipitation events. They systematically concluded that on different temporal and spatial scales (from global to regional scales, and from daily to intra-seasonal scales), precipitation variability has increased across > 75% of the global land area since 1900. In addition, the daily precipitation variability increases by 1.2% per 10 years globally, especially in Europe, Australia, and eastern North America where it is most prominent. They innovatively quantified the increase of warming-related precipitation variability and proposed that it is dominated by anthropogenic greenhouse gas emissions (Fig. 1). The increase of anthropogenic greenhouse gases leads to an increase in atmospheric water vapor content, which favors greater magnitude and variability of precipitation anomalies. They also found that changes in water vapor (thermodynamic factor) contribute more to precipitation variability than changes in atmospheric circulation (dynamic factor). This research was published online in Science, entitled “Anthropogenic Amplification of Precipitation Variability Over the Past Century”.

**Fig. 1:**
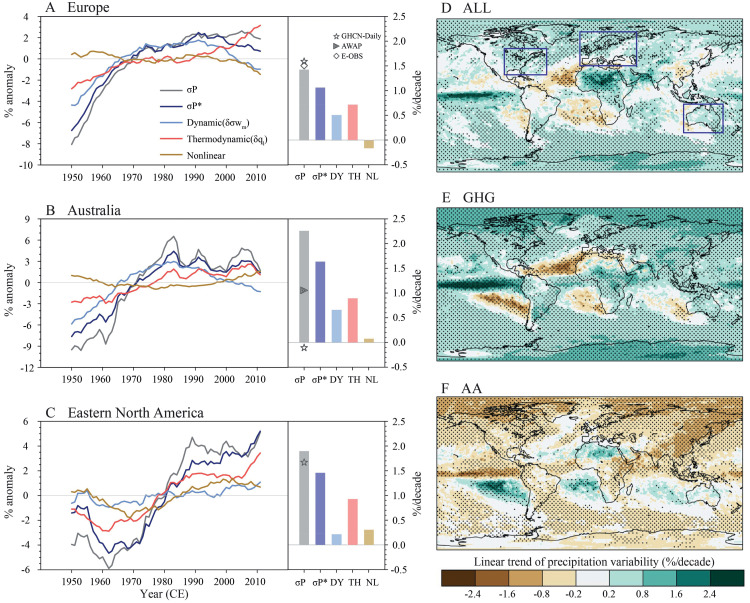
**Physical processes and anthropogenic effects on precipitation variability change (modified from [**1**]).**

The socio-economic impacts of extreme precipitation vary across different regions in the world. In contrast to the monsoon region, the annual precipitation may be determined by a few extreme precipitation events in arid and semi-arid regions, such as arid Central Asia. A single heavy rainfall may cause serious damage to the local economy and the normal life of the people. A significant increase in extreme precipitation has already been reported in arid Central Asia [2,3]. These studies suggest that the increase in extreme precipitation is not driven by the traditionally stable westerly water vapor but by anomalous water vapor from the Indian monsoon and the East Asian monsoon [2,[4], [5], [6]]. Drawing from the research of Zhang et al. [1], it can be considered that anthropogenic greenhouse gas emissions lead to more anomalous water vapor transport from the oceans to the arid Central Asia. This results in increased atmospheric water vapor content, further leading to an increase in extreme precipitation in this region, while internal variability of the climate system primarily affects average precipitation. Additionally, previous research has shown regional differences in the contributions of water vapor changes (thermodynamic factor) and atmospheric circulation changes (dynamic factor) to precipitation. For example, in the arid region of Northwest China, where precipitation is scarce, water vapor changes (thermodynamic factor) have a greater contribution to precipitation. However, in the monsoon region of North China, where precipitation is abundant, the vertical movement (dynamic factor) contributes more to precipitation changes [7]. Therefore, regarding extreme precipitation, does the contribution of water vapor changes (thermal component) dominate across different regions globally? Is there a regional difference similar to that observed for average precipitation? These questions also deserve further investigation in future research.

(A to C) Time series for precipitation variability (σP; gray), vertical moisture advection variability [$\sigma (-\frac{\omega_{m}q_{l}}{g})$, denoted as σP* for brevity; dark blue], dynamic component ($\delta \sigma \omega_{m}$; light blue), thermodynamic component ($\delta q_{l}$; red), and nonlinear component (brown) from 1940 to 2020 are calculated from ERA5 data. These terms are normalized over the entire period and presented as percentage anomaly. Bars indicate the regional average trends for each term over the entire period based on ERA5 (% per decade). Markers show the observed precipitation variability trend over the same period. Results are presented for Europe (A), Australia (B), and eastern North America (C), with the regions highlighted in (D) with blue box. (D to F) Linear trend of precipitation variability (% per decade) over the past century (1900–2020) under all external forcings [ALL, (D)], greenhouse gas forcing [GHG, (E)], and anthropogenic aerosol forcing [AA, (F)]. Shadings show the multimodel ensemble mean trend, with dots indicating where 10 out of 12 models agree on the sign of change.

## Declaration of competing interest

The authors declare that they have no conflicts of interest in this work.
